# Antibody Mediated Immunity to SARS-CoV-2 and Human Coronaviruses: Multiplex Beads Assay and Volumetric Absorptive Microsampling to Generate Immune Repertoire Cartography

**DOI:** 10.3389/fimmu.2021.696370

**Published:** 2021-07-27

**Authors:** Jiong Wang, Dongmei Li, Qian Zhou, Alexander Wiltse, Martin S. Zand

**Affiliations:** ^1^Department of Medicine, Division of Nephrology, University of Rochester Medical Center, Rochester, NY, United States; ^2^Clinical and Translational Science Institute, University of Rochester Medical Center, Rochester, NY, United States

**Keywords:** SARS-CoV-2, human coronaviruses (HCoVs), anti-S and anti-N antibodies, volumetric absorptive micro-sampling (VAMS), mPlex-CoV assay, preexisting human coronavirus immunity, cross-reactive antibody immunity, COVID-19 vaccine studies

## Abstract

The COVID-19 pandemic is caused by SARS-CoV-2, a novel zoonotic coronavirus. Emerging evidence indicates that preexisting humoral immunity against other seasonal human coronaviruses (HCoVs) plays a critical role in the specific antibody response to SARS-CoV-2. However, current work to assess the effects of preexisting and cross-reactive anti-HCoVs antibodies has been limited. To address this issue, we have adapted our previously reported multiplex assay to simultaneously and quantitatively measure anti-HCoV antibodies. The full mPlex-CoV panel covers the spike (S) and nucleocapsid (N) proteins of three highly pathogenic HCoVs (SARS-CoV-1, SARS-CoV-2, MERS) and four human seasonal strains (OC43, HKU1, NL63, 229E). Combining this assay with volumetric absorptive microsampling (VAMS), we measured the anti-HCoV IgG, IgA, and IgM antibodies in fingerstick blood samples. The results demonstrate that the mPlex-CoV assay has high specificity and sensitivity. It can detect strain-specific anti-HCoV antibodies down to 0.1 ng/ml with 4 log assay range and with low intra- and inter-assay coefficients of variation (%CV). We also estimate multiple strain HCoVs IgG, IgA and IgM concentration in VAMS samples in three categories of subjects: pre-COVID-19 (n=21), post-COVID-19 convalescents (n=19), and COVID-19 vaccine recipients (n=14). Using metric multidimensional scaling (MDS) analysis, HCoVs IgG concentrations in fingerstick blood samples were well separated between the pre-COVID-19, post-COVID-19 convalescents, and COVID-19 vaccine recipients. In addition, we demonstrate how multi-dimensional scaling analysis can be used to visualize IgG mediated antibody immunity against multiple human coronaviruses. We conclude that the combination of VAMS and the mPlex-Cov assay is well suited to performing remote study sample collection under pandemic conditions to monitor HCoVs antibody responses in population studies.

## Introduction

The coronavirus infectious disease 2019 (COVID-19) pandemic has taken over 2.5 million lives worldwide, and over 500,000 in the US, as of March 15, 2021 (1). It has become the largest global public health emergency in this century. The causative pathogen is the highly contagious Severe Acute Respiratory Syndrome Coronavirus 2 (SARS-CoV-2), a novel enveloped, positive-sense, single-stranded RNA virus of the coronavirus (CoV) family. Other CoVs have caused human epidemics with severe acute respiratory syndrome (SARS), including SARS-CoV-1 in 2002–2003 (2); the Middle East respiratory syndrome coronavirus (MERS-CoV) in 2012 (3). Other human seasonal α (e.g. 229E, NL63) and β (OC43, HKU1) HCoVs cause mild respiratory infections (4, 5). Of note, the β HCoVs are categorized into several lineages based on genomic similarity: Lineage A (e.g. OC43, HKU1), Lineage B (e.g. SARS-CoV-1, SARS-CoV-2), and Lineage C (e.g. MERS-CoV) (6).

All HCoVs contain four structural proteins: the spike (S), envelope (E) and membrane (M) compose the viral envelope, and the nucleocapsid (N) protein binds the viral genomic RNA (5). Currently, the viral surface homotrimeric glycoprotein S and internal N protein are considered to have the highest immunogenicity (7). The S protein has S1 and S2 subunits. A receptor-binding domain (RBD) on the N-terminal S1 subunit has high affinity for host cell surface angiotensin-converting enzyme 2 (ACE2) and mediates viral entry, while the S2 subunit is responsible for virus-cell membrane fusion (8). Emerging evidence suggests that ACE2-blocking monoclonal antibodies may protect against SARS-CoV-2 infection in animal models (9). Also, the clinical studies showed that the convalescent plasma transfusion may reduce mortality in critically ill patients and showed beneficial effect on clinical symptoms (10).

In-depth serologic analyses are essential for understanding the prevalence of, and immunity to, SARS-CoV-2. Recently, a number of antibody binding assays based on the S or N antigens have become available, primarily enzyme-linked immunosorbent assays (ELISAs) (11) and lateral flow assays (LFAs) (12), as well some Luminex assay based multiplex assays (13–16). However, these assays are primarily focused on the SARS-CoV-2 S and/or N proteins, and not the broad range of CoV Cross-reactivity.

Multiplex systems serology assays that measure IgG binding for multiple antigens have been used to quantify antigenic distances between viral strains, track antibody cross-reactivity due to prior IgG exposure to similar viral strains, and provide quantitative measurements of IgG repertoire changes after infection or vaccination (17). We have previously described an influenza anti-hemagglutinin multiplex assay, mPlex-Flu, that has a continuous linear readout over 4.5 logs, and low Type-I (false positives, specificity) and Type-II (false negatives, sensitivity) errors (18–20). The mPlex-Flu assay provides absolute concentrations of antibodies against up to 50 target analytes (17, 21–23) with extremely low inter- and intra-assay variance, greater precision of clinical trial group statistical comparisons (20, 24), and a very high correlation with functional viral binding and inhibition assays. It allows for rapid characterization of the similarity between the dominant antigens of disparate influenza viral strains. Notably, the mPlex-Flu assay requires! 5 μL serum or plasma, and can be used with 10 μL samples obtained by fingerstick capillary blood volumetric absorptive micro-sampling (VAMS) (18). These characteristics overcome several major translational barriers to remote sample collection for assessment of antibody-mediated immunity. Thus, such an assay would be highly desirable to track antigenic similarity between emerging SARS-CoV-2 variants, and the evolution of vaccine or post-infection immunity, and to guide vaccine development.

**Figure 1:**
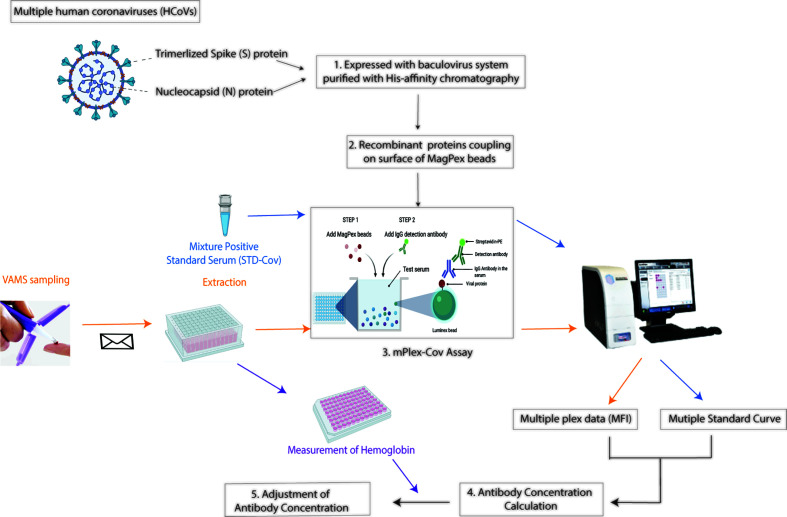
The study workflow. 1. Expression of recombinant spike (S) proteins and nucleocapsid (N) of Human coronavirus (HCoVs) listed in **Table 1** with baculovirus expression system. The proteins were then purified with His-affinity chromatography. 2. Coupling the individual recombinant proteins of HCoVs onto fluorescence-coded Luminex beads. 3. Establish the multiple plex assay, mPlex-Cov. 4. Calculation of HCoV strain-specific antibody concentration with the standard curve of each individual virus strain. 5. Adjustment of antibody concentration in the blood sample by hemoglobin to correct for the actual serum volume. (Figure was generated with BioRender.com).

In this report, we describe the combination of VAMS and a 15-antigen multiplex mPLEX-CoV assay for simultaneous measurement of IgG, IgA, and IgM against the S and N proteins of SARS-COV-1, SARS-CoV-2 and four seasonal HCoVs (OC43, HKU1, NL63, 229E), as well as the S1, S2, and RBD protein domains of SARS-CoV-2. The assay is designed specifically to detect cross-reactivity of serum antibodies, and for ease of longitudinal sampling with only a fingerstick blood sample. Using a HCoV standard serum we are able to determine serum IgG, IgA, and IgM concentrations, and thus compare absolute antibody levels against different HCoVs (24). Here we validate the accuracy and reproducibility of VAMS combined with the mPlex-CoV assay (**Figure 1**). We then demonstrate a clear multidimensional separation between the pre-COVID, COVID, and COVID-vaccine groups in their IgG using metric multidimensional scaling (MDS) analysis of the measured IgG concentrations. This proof-of-concept study demonstrates that fingerstick VAMS with the mPlex-CoV assay would significantly improve our ability to study the role of preexisting HCoV antibodies on SARS-CoV-2 infection and vaccination immune responses, and to conduct remote sample collection and multidimensional systems serology in COVID-19 population immunity studies and clinical trials. This will become increasingly important as newer SARS-CoV-2 variants emerge.

**Table 1:** Gene sequences of HCoV spike and nucleocapsid proteins.

Gene Bank Strain Name	Abbrv	Gene Bank Accession #	S protein size (aa)	N protein size (bp)
SARS-CoV-2	SARS2	EPI_ISL_402130	1287	419
SARS-Cov-1	SARS1	NC_004718.3	1256	433
MERS	MERS	JX869059	1354	413
HCoV-HKU1	HKU	AY597011	1357	441
HCoV-OC43	OC43	KX344031.1	1359	448
HCoV-NL63	NL63	NC_005831.2	1293	378
HCoV-229E	229E	KY369911.1	1172	389

## Material and Methods

### Human Subjects Protection

This study was approved by the Research Subjects Review Board at the University of Rochester Medical Center (RSRB approval numbers STUDY00004836, STUDY00005001, STUDY00001185). All subjects were consented to this study for blood sample usage, and all subject data were coded such that subjects could not be identified, either directly or through linked identifiers.

### Participants and VAMS Sample Collection

#### Pre-COVID Cohort

VAMS fingerstick samples (10 μL) were collected before January 2020 from 18 to 65-year-old healthy volunteers as previously described (18). Subjects taking immunosuppressive medications were excluded. Subjects (n=21) included 12 females, 9 males; and the median age was 56.

#### SARS-CoV-2 Antibody-Positive Cohort

VAMS fingerstick samples (10μL) were collected from (1) eighteen COVID-19 convalescent subjects who had previously been diagnosed with COVID-19 by RT-PCR assay, which included 12 females and 6 males, median age was 53. (2) Fourteen post-vaccination subjects who have received two doses of Pfizer COVID-19 vaccination, including 8 females and 6 males with a median age 57. The subjects’ information is also listed in **Supplementary Table S1**. All VAMS tips were stored in sealed containers with silica desiccant packets at -20°C until analysis.

### Human Coronavirus Positive Control Standard Serum (STD-CoV)

For a positive control standard, we used a mixture of equal volumes from four serum specimens selected from a COVID-19 positive cohort collected in April 2020 from 140 unique patients (13). These sere were selected for high IgG titers against SARS-CoV-1, SARS-CoV2, HCoV-OC43, HCoV-HKU1, HCoV-NL63, and HCoV-229E. The total IgG, IgA, and IgM immunoglobulin concentration in the serum were estimated to be 6.24 ± 0.17 mg/mL, 2.75 ± 0.28 mg/mL, and 0.40 ± 0.04 mg/mL, respectively, using the Millplex@MAP human immunoglobulin isotyping multiplex assay kit (MilliporeSigma, Germany, Cat# HGAMMAG-301K).

### Recombinant HCoV Spike (S) and Nucleocapsid (N) Protein Expression

**Figure 2:**
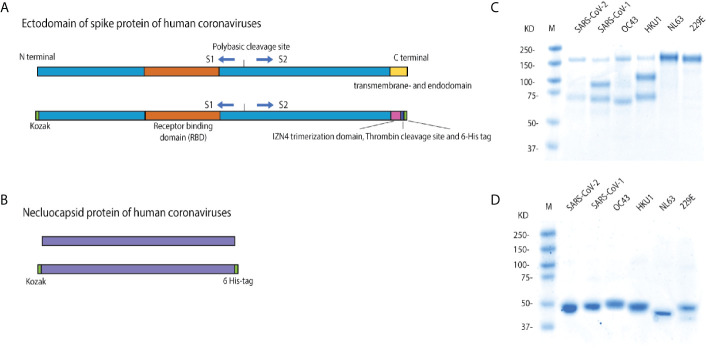
Constructs for recombinant spike and nucleocapsid protein expression. **(A)** Schematic of the wild type HCoV full length spike protein (S), with the ectodomain, receptor binding domain, furin cleavage site, S1, S2, and transmembrane and endodomain domains indicated; and the soluble trimeric spike. In the protein constructs, the transmembrane and endodomain were replaced with a region containing an IZN4 trimerization domain, a thrombin cleave site and a 6X-hexahistidine tag. **(B)** Schematic of the construct of nucleocapsid (N) proteins of human coronavirus strains. **(C)** Reducing SDS-PAGE gel of the expressed and purified, soluble, and trimerized HCoV spike proteins. These proteins have a furin-like cleavage site, similar to the related β HCoVs (26–28). While the proteins have multiple bands under reducing conditions, both bands were pulled down with Ni-NTA His-tag affinity beads, indicating that both cleaved subunits are still bound together in native conditions, but disassociate in the denaturing conditions. **(D)** The reducing SDS-PAGE gel of the expressed and purified HCoV nucleocapsid proteins.

We expressed and purified recombinant stabilized trimeric S and N proteins from multiple HCoV strains. The protein encoding codon-optimized nucleotide sequences for each protein (**Table 1**) were synthesized commercially by Integrated DNA technology (IDT). We expressed the S protein ectodomains only, without the transmembrane and endo-domains. Fusion S protein genes were constructed with an N terminal Kozak motif and a C-terminal IZN4 fold on trimerization domain that offers less antigenicity than T4 trimerization after incubation of mice (25), and a thrombin cleavage site. Both S and N proteins had an added C-terminal hexahistidine tag (**Figures 2A, B**). S and N protein constructs were cloned into the pFastBac baculovirus expression vector (Invitrogen), and expressed as previously described (17, 21). Recombinant proteins were then purified using Ni-NTA Agarose affinity columns (Qiagen) (17, 21). Each protein was concentrated and buffer-exchanged with Amicon centrifugal units (EMD Millipore) in phosphate-buffered saline (PBS) with 1X Protease Inhibitor Cocktail (ThermoFisher, Cat No: 78430), and stored at -80°C. Protein purity was analyzed by SDS-PAGE (**Figures 2C, D**).

### Multiplex Bead Coupling

Purified trimerized S and monomer N proteins were coupled to magnetic microsphere beads (Luminex, Austin, TX) according to the manufacturer’s protocol using the xMAP^®^ Antibody Coupling (AbC) kit (Luminex, Austin, TX). All S and N proteins were coupled at a concentration of 40 pmole/10^6^ beads. Uncoupled sites were blocked with phosphate-buffered saline (PBS) pH7.4 containing 1% bovine serum albumin (BSA). Protein-coupled microspheres were washed with PBS and stored in storage buffer (PBS pH7.4 + 0.01% BSA) at +4°C in the dark until use. For analysis of SARS-CoV-2 subdomain binding, we coupled commercial monomeric SARS-CoV-2 S1+S2, S1, RBD and S2 proteins (SinoBio, China) to beads as above (**Figure 2**).

### Antibody Reagents

The HCOV protein coupled beads were tested and validated with commercial monoclonal and polyclonal antibodies against S- and N- proteins of HCoVs (SinoBiological Inc; Antibodies-Online; EMD Millipore; see details listed in **Supplementary Table S2**). Secondary antibodies were PE-conjugated anti-human, rabbit and mouse IgG antibodies from Southern Biotech (Birmingham, AL).

### VAMS Sampling

Fingerstick blood samples were collected using a volumetric absorptive microsampling (VAMS) 10μL device (Mitra Collection Kit; Neoteryx, CA, USA) following the manufacturer’s instructions (18). Each of two tips absorbed 10μL of capillary blood, for a total of 20μL of blood per collection. Immediately after sampling, all VAMS tips were placed in sealed containers with silica desiccant packets, and stored at –20°C until analysis. To extract the antibodies, VAMS tips containing 10μL blood samples were individually soaked in 200μL extraction buffer (PBS + 1% BSA + 0.5% Tween) in 1 mL deep well plates (Masterblock, GBO, Austria) and shaken overnight as previously described (18). The eluant was stored at 4°C until analysis.

### mPlex-CoV Assay

The mPlex-CoV assay was performed as described previously (17, 21, 23). 200μL of eluent from VAMS devices (1:20) was further diluted 1:50 to yield a final sample dilution of 1:1,000. For analysis, 50 μL each of diluted sample was added to black, clear-bottom 96 well plates (Microplate, GBO, Austria) in duplicates. 50μL of the bead panel mixture (**Table 2**), containing the coupled HCoV S- and N- proteins, was added to each well of the plate as previously described (17, 21, 23). Plates were then incubated with gentle shaking for 2 hours at room temperature, and then washed [PBS + 1% Brij 35 (Thermo Scientific, IL, USA) + 0.1% BSA] while immobilized with a magnet placed under the plate. After three washes, a goat anti-human PE-conjugated IgG, IgA and IgM secondary antibody (Southern Biotech, Cal No:2040-09, 2050-09, 2020-09, respectively) was added, and plates were incubated for another 2 hours. After three more washes, the beads were re-suspended in drive fluid (Luminex Co., TX) and IgG binding analyzed using MAGPIX™ Multiplex Reader (Luminex Co., TX). The calculation of IgG antibody concentrations against each individual HCoV virus strain was performed using the Bio-Plex Manager™ 6.2 software (Bio-Rad Co., CA).

**Table 2:** The mPlex-CoV assay panel of human coronaviruses.

Type	Coronavirus	Protein	Abbreviation	Intra-assay	Inter-assay	ULOQ	LLOQ
			region	CV(%)	CV(%)	(ng/ml)	(ng/ml)
β	SARS-CoV-2	S	SARS2	3.24±3.53	3.33±3.12	3324.05±178.76	8.34±3.30
	SARS-CoV-1	Protein	SARS1	6.27±5.02	8.64±6.49	834.70±2.02	6.74±3.99
	HCoV-OC43		OC43	4.16±3.12	4.95±4.54	4109.26±137.54	3.65±1.72
	HCoV-HKU1		HKU1	4.00±3.12	6.00±5.61	523.06±5.88	0.70±0.20
α	HCoV-229E		229E	3.22±2.88	5.84±5.87	466.25±1.24	0.64±0.08
	HCoV-NL63		NL63	3.32±2.93	6.54±5.33	266.24±2.22	0.77±0.56
β	SARS-CoV-2	N	SARS2	3.22±2.10	4.81±4.96	2123.34±956.08	3.09±2.54
	SARS-CoV-1	Protein	SARS1	1.93±2.39	4.52±4.06	228.87±0.55	0.73±0.54
	HCoV-OC43		OC43	3.41±2.13	8.76±6.29	584.80±15.16	0.83±0.07
	HCoV-HKU1		HKU1	6.92±1.17	6.86±5.07	138.33±0.51	0.60±0.06
α	HCoV-229E		229E	1.43±1.49	6.42±4.75	125.23±1.46	0.27±0.15
	HCoV-NL63		NL63	2.72±1.89	5.71±6.40	365.89±0.56	1.02±0.80
β	SARS-CoV-2	RBD	RBD*	3.62±2.79	3.47±3.43	6538.72±194.67	13.58±2.75
		S1	S1*	3.70±2.93	5.44±5.19	2475.69±43.50	2.18±1.88
		S2	S2*	2.88±2.07	4.81±4.74	2408.97±88.60	6.95±2.35

RBD, Receptor binding domain of SARS-CoV-2 S protein.

*Reagents purchased from SinoBiological. S1+S2 (40589-V08B1), RBD* (40592-V08H), S1 (40591-V08H), S2 (40590-V08B).

The ULOQ and LLOQ were determined by four-fold serial dilution of STD-HCoV serum starting at 1:1000.

### Measurement of Hemoglobin (Hgb) and Adjustment Antibody Concentration

It was necessary to correct IgG, IgA, and IgM concentrations for plasma volume, as the VAMS method collects capillary (i.e. unfractionated) blood, as previously described (18). This allows direct comparison of Ig concentrations with serum samples. We measured hemoglobin concentrations using a bi-colorimetric assay (Abcam, Cat No:ab234046, MA, USA). Briefly, 20μL each of extracted VAMS samples were incubated with 180μL of hemoglobin detector buffer at room temperature for 15 minutes in 96 well plates. The absorbency at 575 nm (*OD*_575_) was measured using a Synergy Microplate reader (BioTek, VT, US), and converted to each isotype antibody concentration using a standard curve calculated by linear regression of six known hemoglobin standards (18). The hematocrit (cellular fraction) of the VAMS sample was estimated from the Hgb concentration and used to estimate the serum HCoVs IgG concentration as described previously (18):

(1) $$\left[IgG_{Serum}\right]=\frac{-31.44\times [IgG_{VAMS}]}{Hgb-35.85}.$$

### Five Parameter Logistic Curve Estimation of Antibody Concentration

We estimated the concentrations of HCoV S or N IgG antibodies in each sample with the standard curve of each analyte generated with eight 1:4 serial dilutions of STD-CoV serum as previously described (17, 20, 21). Duplicates of 50μL diluted STD-CoV were mixed with 50μL of the HCoV bead panel mixture containing 1,000 coupled beads per analyte. After washing and incubating with a secondary antibody, the fluorescence intensity was read using a MAGPIX™ Multiplex reader. A 5-parameter logistic model was then fitted to the data:

$$MFI=d+\frac{\left(a-d\right)}{(1+{\left(\frac{\left[IgG\right]}{c}\right)}^{b})g}$$

where *MFI* = median fluoresce intensity, [*Antibody*] = the antibody concentration, and *a, b, c, d, g* are free parameters estimated from the data. This model was fitted to multiplex measurements for each analyte from serially diluted sera using the method of weighted least-squares. Antibody concentrations were then estimated by inverse regression.

### Estimating Variation of the mPlex-CoV Assay Measurements

The lower limit of detection (LLOD) was defined as the mean plus two standard deviations of the assay blank, which consisted of HCoV S- or N- protein coupled beads incubated with PBS only. The upper limit of detection (ULOD) was the highest value on the linear portion of the 5 parameters logistic curve within 20% accuracy. The lower and upper limits of quantification (LLOQ and ULOQ) for each HCoV S and N isotype antibody analyte were calculated by Bio-Plex Manager 6.1.1 software. Results are shown in the report section. We calculated the mean of ULOQ and LLOQ values generated from three independent assays. Then, the mean of ULOQ and LLOQ for each HCoV S and N IgG, IgA, and IgM antibody were defined and used for the working range of the assay.

The intra- and inter-assay variations for the HCoV multiplex assay were determined as previously described (21). Briefly, intra-assay variability was estimated by testing seven sera in triplicates and calculating the percentage coefficient of variation (CV) between triplicates, and the average CV of seven serum samples. The inter-assay variation was determined by running three independent assays of the same seven sera. The percentage CV was calculated as the difference between three repeat assays for each serum sample, then averaging the CV of the seven samples.

### Statistical and Quantitative Analysis

For group comparisons, all statistical analysis was performed with SAS v9.4 (SAS Institute, Inc., Cary, NC) and R version 3.5.1 software. The Mann-Whitney test was used to test differences in the mean anti-S or anti-N IgG concentrations between pre- and post-COVID groups for each strain. The significance level for all statistical tests was set at *p* = 0.05. Data visualizations were performed using Mathematica (version 12; Wolfram Research, Inc.). Immune repertoire cartography was performed as previously described (18, 21, 29). IgG, IgA, and IgM reactivity against the panel of HCoV was expressed as a 7- (S protein) or 6- (N protein) dimensional coordinate vector, and an n-dimensional Euclidean distance calculated. Metric multidimensional scaling was used to project subject reactivity into a 2-dimensional space, preserving the immune repertoire distance between subjects.

## Results and Discussion

### Control Antibody Binding to HCoV N and S Proteins Assessed by Multiplex Assay

There is accumulating evidence that preexisting IgG against seasonal HCoVs plays a role in the immune response to SARS-CoV-2 infection (30, 31), which is the motivating factor behind our development of an accurate assay to estimate antibodies against multiple strains of HCoVs. We therefore adapted our multiplex antibody assay (18, 24) to detect antibodies that react to 12 HCoV S and N proteins, as well as antibodies that recognize the subunits of SARS-CoV-2, S1, S2 and RBD. Purified HCoV S and N proteins were coupled to fluorescent coded microsphere beads at equimolar concentration (17).

**Figure 3:**
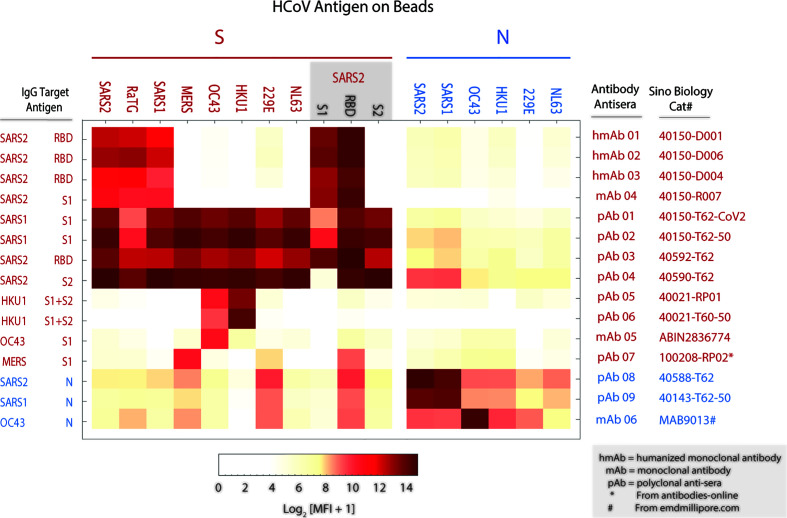
Verification of mPlex-Cov assay: The binding profile (MFI) of spike (S) and nucleocapsid (N) proteins coupled to Luminex beads reacting with monoclonal and polyclonal antibodies. The antibodies were serially diluted based on the concentrations recommended by manufacture manual. The heatmap displays the average median florescence intensity (MFI) of duplicate wells using the representative dilution for each antibody listed. See Methods for specific antibody catalog numbers and suppliers.

To assess the efficiency and specificity of mPlex-CoV assay to detect the SARS-CoV-2 specific antibodies, we first measured IgG antibody binding with a panel of rabbit anti-HCoV-S and -N polyclonal antisera and monoclonal IgG antibodies derived from SARS-CoV-2 S antigen vaccination using the mPlex-CoV assay (**Figure 3**). IgG reactivity against the SARS-CoV-2 S protein was most specific with the monoclonal antibodies generated by the animal immunized with purified SARS-CoV-2 RBD protein (see **Supplementary Table S2**). As expected, these antibodies also had high cross-reactivity with the Lineage B CoV SARS-CoV-1, and with the bat CoV strain RaTG13 that has high SARS-CoV-2 sequence homology (32). The SARS-CoV-2 spike protein gene sequence is most similar to SARS-CoV-1, having 90% homology in N and 76% homology in S. In contrast, the anti-SARS-CoV-2 polyclonal rabbit sera raised against either the S1 or RBD domains were broadly cross-reactive against all HCoV S proteins including the pha (NL63, 229E) and β (OC43, HKU1, MERS) HCoV spike proteins. This was somewhat striking given that the SARS-CoV-2 S protein sequence homology to other β HCoVs is only ~29%, and ≤23–25% to the two α HCoVs (33). This finding may potentially be explained by the higher homology between the S2 subunits of all HCoVs (30, 31). In contrast, polyclonal rabbit sera raised against the MERS spike protein S1 region had minimal cross-reactivity against the SARS-CoV-2 spike S1 region, with some reactivity against the SARS-CoV-2 RBD. These evaluations were somewhat constrained by the limited availability of commercial polyclonal antisera and monoclonal antibodies that specifically recognize the S and N proteins of seasonal HCoVs β-HCoV (OC43, HKU1) and the α-HCoV (NL63, 229E).

Anti-N protein IgG raised against SARS-CoV-2 and SARS-CoV-1 had modest cross-reactivity against β-HCoV (OC43, HKU1) N proteins, and to a lesser extent the α-HCoV (NL63, 229E) N proteins. This is consistent with reports that monoclonal antibodies raised against SARS-CoV-2 N protein regions have minimal reactivity against α and β-HCoVs (34). In addition, these polyclonal anti-N protein antisera also demonstrated faint non-specific binding with HCoV S proteins, such as SARS-CoV-2 RBD and 229E proteins. Interestingly, a recent clinical study showed that SARS-CoV-2 infection elicited the N-reactive IgG antibody also cross-react with the N protein of seasonal HCoVs, with more substantial cross-reactivity, with - greater than β-HCoV reactivity despite having less sequence homology (35).

Taken together, these results confirmed the successful coupling of the target proteins to the multiplex beads, and that the expressed and purified S- and N- proteins were bound by both monoclonal antibodies and anti-SARS-CoV-2 polyclonal antisera.

### Quantifying Human Anti-HCoV S and N IgG, IgA, and IgM

**Figure 4:**
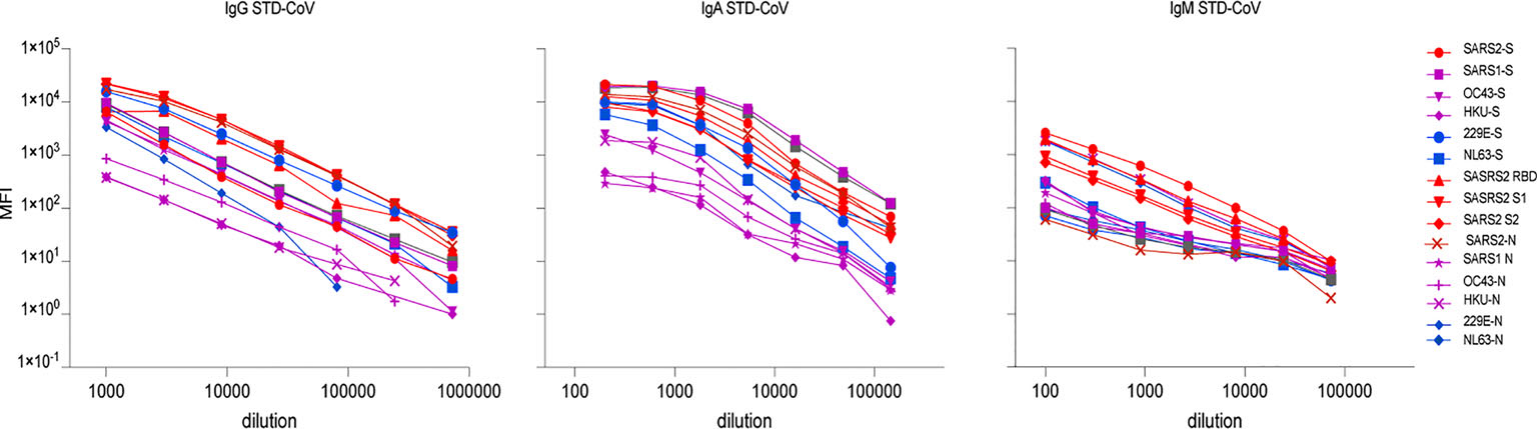
The standard curves of IgG, IgA, and IgM antibody of the individual spike (S) and nucleocapsid (N) proteins of control positive serum of human coronaviruses (STD-CoV). A polyclonal coronavirus standard (STD-CoV) anti-serum of IgG, IgA, and IgM used was a mixture of four anti-SARSCoV-2 positive sera, which contained high antibodies against the SARS-CoV-2 and some of seasonal human coronaviruses. Each spot represents the mean concentration of antibody against one strain of HCoVs from duplicate wells.

Using the anti-HCoVs positive control standard serum (STD-CoV) in mPlex-CoV assay, we next generated 5 parameter logistic curves to map the assay MFI results to antigen-specific antibody concentrations with the method previously described for influenza anti-hemagglutinin antibodies (19–21). This allows direct comparison of absolute individual isotype antibody concentrations against each target strain HCoV S or N protein. In brief, a standard, pooled, positive control serum (STD-CoV) was created with high levels of anti-S and N IgG against all the analytes by mixing sera from 4 different subjects, and the standard curve shown in **Figure 4**. We then assayed serial dilutions of this serum reacting with anti-human IgG+IgA+IgM Fc specific capture antibody (Sigma-Aldrich, MO, USA) to create the total IgG, IgA, and IgM standard curves. STD-CoV at the same dilutions was then used to generate individual standard curves for each of the 15 HCoV antigens (**Figure 4**). Parameters for a 5-parameter logistic regression model, relating MFI to antibody concentration, were estimated from duplicate measurements and 3 independent assays for each HCoV antigen with Bio-Plex Manager™ 6.2 software. As previously noted for influenza anti-HA IgG binding, there are expected between-strain differences for each standard curve, which can cause slight variations in the density of the different HCoV antigen coupled multiplex beads. This method allows absolute IgG, IgA and IgM concentrations for each analyte to be measured over a 4.5 Log_10_ range (**Figure 4**). A limitation of this approach for IgA and IgM antibody quantification was the low levels of anti-N IgG and IgA antibodies to OC43 and HKU1 and anti-N IgM antibodies to most HCoVs viruses (**Figure 4**), which may increase false discovery rates during the concentration calculation for IgA and IgM; this was not an issue for IgG.

### mPlex-CoV Assay Has Low Intra- and Inter-Assay Variability

Another benefit of fluorescent multiplex assays is their ability to generate with low inter- and intra- sample variability. To verify this property of the mPLEX-CoV assay, we conducted three independent mPlex-CoV measurements with serially diluted STD-CoV samples. Using these results, we calculated the lower and the upper limits of quantification (LLOQ and ULOQ) for each HCoV S and N IgG antibody and the percentage intra- or inter-coefficient of variation (CV) as described above (**Table 2**). All intra-assay CVs were <~7%, and inter-assay CVs <~9%. The parameters of IgA and IgM mPlex-CoV assay are listed in **Supplementary Tables S3 **and** S4**, respectively.

One important finding from these quality measurements is that we observed significant decreases in MFI when the serum was diluted less than 1:800 for IgG, or 1:100 for IgA mPlex-CoV assay. While the mechanism of this was unclear, we therefore selected an initial serum dilution of 1:1000 for IgG and 1:200 for IgA to test all serum samples, including those used to calculate the ULOQ and ULOQ values. With these dilutions, we found that the 4-log range of standard curves of anti-SARS2 and SARS1 S and N IgG, including the ULOQ, could reach >6,000 ng/ml for anti-SARS2-RBD IgG. We also found that serum IgG concentrations against seasonal HCoVs were significantly lower than post-infection anti-SARS2 IgG, and thus the anti-seasonal HCOV levels in the control STD-CoV serum were also lower than those of post-infection anti-SARS2 IgG. This was true even when serum samples with high-level antibodies against other HCoVs were assayed. These results are consistent with our previously published SARS2 antibody clinical study (13).

### Antibody Concentration Calculations for VAMS Samples

**Figure 5:**
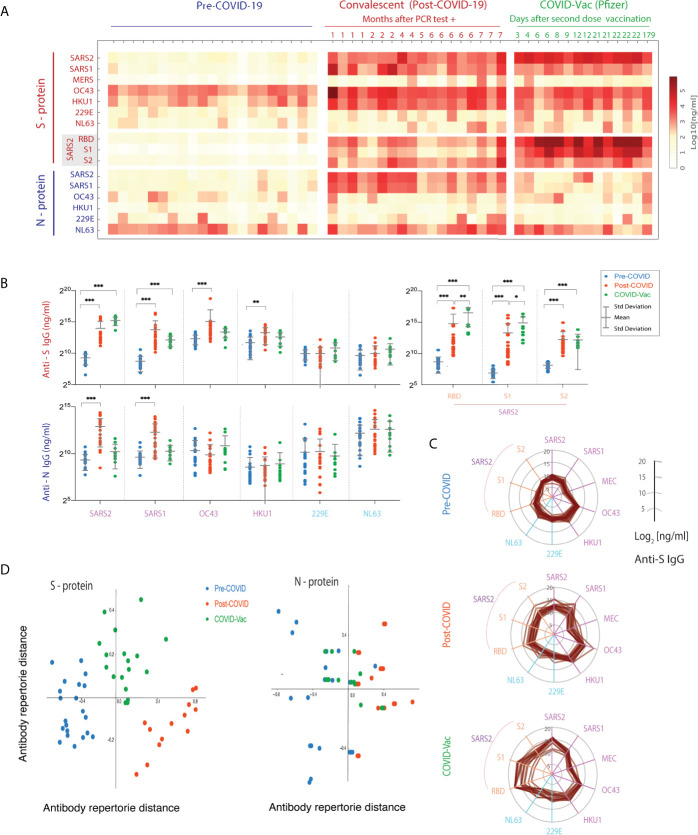
Spike (S)- and nucleocapsid (N)-reactive IgG antibody levels to human coronaviruses (HCoVs) in VAMS blood samples of pre-COVID, convalescents, and post-vaccination subjects. **(A)** The IgG concentrations (ng/mL) of each S and N-reactive IgG antibodies against HCoVs. The heatmap shows mean concentration (ng/ml) of duplicate samples. **(B)** Comparison of differences in range of anti-HCoV S- and N- protein IgG between pre-, post-COVID and post-vaccination cohorts. (***p < 0.001, **p < 0.01,*p < 0.05). **(C)** The broad cross-reactive anti-S IgG antibody concentrations of HCoVs in three cohorts shown in multivariate spider plots, with each axis representing log2 IgG concentrations. **(D)** Multidimensional Scaling (MDS) analysis of the VAMS results.

One advantage of the VAMS method is the safety and simplicity of the process (27). We have verified that finger-stick whole blood sampling combined with Hgb measurement to estimate serum IgG antibody concentrations quantitatively is highly consistent with traditional serum sampling methods (18). Additionally, results from finger-stick micro-sampling at home are highly consistent with finger-stick sampling performed on-site. In this study, we collected capillary blood from 21 pre-COVID-19and 18 convalescent subjects using VAMS. Serum fraction was estimated using the Hgb concentration, as previously described (18). The anti-HCoV IgG results are shown in **Figure 5**, and those of IgA and IgM in **Supplementary Figure S1**.

In the pre-COVID cohort, we found α and β HCoV S- and N-reactive IgG antibodies present in samples from 18-65 year old healthy donors. This is consistent with the previously reported prevalence of OC43, HKU1, and NL63 infection in adults and children (5, 36, 37). In contrast, VAMS samples from COVID convalescent subjects >28 days post-infection demonstrated increased levels of anti-S- and N protein IgG, IgA, and IgM for specific SARS-CoV-2 and SARS-CoV-1 virus strains, as well as significantly increased anti-OC43 and HKU1 S-reactive IgG, IgA and IgM antibody concentrations compared to the pre-COVID cohort. However, the increased N-reactive antibody levels were only observed in the post-COVID cohort against SARS-CoV-2 and -1 viruses, whereas no anti-N IgG changes were observed against other seasonal HCoVs in the post-COVID and post-vaccination cohorts. Notably, the COVID-19 convalescents showed significantly higher anti-S and N IgM antibodies to SARS-CoV-2 within the first two months (**Supplementary Figure S1**). We also note that the concentrations of NL63 anti-N were higher than those for anti-S levels. The reasons for this finding are not immediately apparent. There is very limited data regarding the persistence of NL63 N-reactive IgG and IgA antibody, or comparisons between anti-N and anti-S levels.

Importantly, the pre-COVID adult control subjects had essentially undetectable levels of SARS-CoV-2 S- and N-reactive IgG, IgA, and IgM antibodies. One might expect bi-directional S- and N-reactive antibody cross-reactivity between OC43, NL63, and SARS-CoV-2 given their close protein sequence homologies, including the conserved S protein RBD and S2 subunits. In fact, a high level of cross-reactivity between S- and N-reactive IgA to HCoVs viruses was shown (**Supplementary Figure S1**), and there are notable cross-reactions of N-reactive IgA and IgM antibodies to SARS-CoV-2 virus and the RBD subdomain. This finding is consistent with a recent study that showed cross-reactivity of N-reactive antibodies between NL63 and SARS-CoV-2 (35).

We also found that, compared to pre-COVID controls, the post-vaccination cohort showed statistically significant increases in anti-SARS-CoV-2 S-specific IgG, IgA, and IgM antibodies (**Figures 5A, B**). These increases were associated with high levels of anti-RBD and anti-S1 binding IgG, IgA and IgM. Interestingly, the anti-RBD and S1 IgG levels were also statistically significantly higher than longer-term persistent levels seen in the post-infection cohort (**Figure 5B**). One potential mechanism for this observation may be that the vaccination group experienced both priming and boosting immune exposures, while the post-COVID subjects essentially experienced only the priming event (i.e. the initial infection). In addition, it has been reported that SARS-CoV-2 can disrupt B cell responses (38). It remains to be seen if an increase in anti-SARS-CoV-2 RBD specific IgG antibodies will be seen in post-COVID subjects after receiving their first vaccination or if re-infected, both of which could serve as “boosting” immunological events.

To visualize the changes in the anti-S immune repertoire against all HCoVs, we plotted IgG concentrations against individual HCoV strains in the three cohorts with parallel axes polar plots. This enabled us to visualize the profile of anti- β HCoV antibodies post-COVID-19, and the more centralized IgG antibody response to the SARS-CoV-2 and S1 and RBD domains of the spike protein. Note both the increase in IgG concentration after infection and vaccination, and the marked increase in anti-SARS-CoV-2 IgG levels after vaccination. Given the potential cross-reactivity of Ig between HCoV strains, we were also interested to see if pre-COVID, post-COVID and post-vaccination groups could be graphically separated by immune repertoire distance. The distance was calculated using the reactivity against the 7 HCoV S- and N- proteins, and projected onto 2 dimensions using metric multi-dimensional scaling (MDS). Metric MDS analysis was then performed using the Person Correlation distance to project multidimensional IgG concentration results for each subject onto 2 dimensions, reflecting the overall relative immune HCoV S- or N- protein IgG repertoire (**Figure 5D**). This distance metric was selected to distinguish between groups where increases in IgG binding to spike proteins increased or decreased in a similar fashion. The results showed good separation of the three groups (pre-COVID, COVID, COVID-vaccine) for the S, but not N, proteins. This suggests differences in cross-reactivity induced by SARS-CoV-2 infection versus vaccination that affect the human CoV immune repertoire among subjects. The clinical significance of this phenomenon is unclear, and will require further investigation.

## Conclusions

Collecting the blood samples from SARS-CoV-2 infected patients in the community is difficult, especially during the early days of infection, when an individual may be asymptomatic or mildly symptomatic, but still have high potential for transmission. VAMS provides a highly effective remote sampling approach for such situations. The combination of VAMS and mPlex-CoV has the advantage of being able to combine of fully remote enrollment and sampling for clinical COVID-19 studies with low cost, and high accuracy. Such studies will be critical for understanding the humoral immune response to SARS-CoV-2 infection. The combination of capillary VAMS sampling addresses a significant translational barrier in SARS-CoV-2 research and population health studies (18): the ability to remotely collect and ship samples for IgG measurement. Here we extend this approach to the mPlex-Cov assay, using samples obtained by VAMS, and report results from simultaneous assessment of anti-S and -N IgG, IgA, and IgM concentrations against the six most common HCoV strains. In addition, we used MDS analysis to visualize an individual’s IgG mediated immune repertoire against multiple HCoVs, a method we refer to as immune repertoire cartography.

## Data Availability Statement

The original contributions presented in the study are included in the article/**Supplementary Material** (DOI: 10.6084/m9.figshare.13490166), further inquiries can be directed to the corresponding author.

## Ethics Statement

The studies involving human participants were reviewed and approved by The Research Subjects Review Board of the University of Rochester. Written informed consent was obtained from all subjects in this study for blood sample usage.

## Author Contributions

JW and MZ conceived of the investigation plan and designed the experiments and analytic methods. JW, QZ, and AW carried out the experiments. JW, DL, and MZ analyzed the data. JW, MZ, QZ, DL and AW wrote and edited the manuscript. All authors contributed to the article and approved the submitted version.

## Funding

This work was supported by the National Institutes of Health Institute of Allergy, Immunology and Infectious Diseases grant R21 AI138500 and R01 AI129518 (MZ, JW), and the University of Rochester Clinical and Translational Science Award UL1 TR002001 from the National Center for Advancing Translational Sciences of the National Institutes of Health (DL, MZ). The content is solely the responsibility of the authors and does not necessarily represent the official views of the National Institutes of Health. None of the above funders had any role in study design, data collection and analysis, decision to publish, or preparation of the manuscript.

## Conflict of Interest

The authors declare that the research was conducted in the absence of any commercial or financial relationships that could be construed as a potential conflict of interest.

## Publisher’s Note

All claims expressed in this article are solely those of the authors and do not necessarily represent those of their affiliated organizations, or those of the publisher, the editors and the reviewers. Any product that may be evaluated in this article, or claim that may be made by its manufacturer, is not guaranteed or endorsed by the publisher.

## References

[1] WHO. WHO Coronavirus Disease (COVID-19) Dashboard. Report. (2021). https://covid19.who.int/

[2] Rota PA Oberste MS Monroe SS Nix WA Campagnoli R Icenogle JP. Characterization of a Novel Coronavirus Associated With Severe Acute Respiratory Syndrome. Science (2003) 300:1394–9. 10.1126/science.1085952 12730500

[3] Al-Tawfiq JA Zumla A Memish ZA. Coronaviruses: Severe Acute Respiratory Syndrome Coronavirus and Middle East Respiratory Syndrome Coronavirus in Travelers. Curr Opin Infect Dis (2014) 27:411–7. 10.1097/QCO.0000000000000089 25033169

[4] Cui J Li F Shi ZL. Origin and Evolution of Pathogenic Coronaviruses. Nat Rev Microbiol (2019) 17:181–92. 10.1038/s41579-018-0118-9 PMC7097006 30531947

[5] Lim YX Ng YL Tam JP Liu DX. Human Coronaviruses: A Review of Virus-Host Interactions. Diseases (2016) 4:2079–9721. 10.3390/diseases4030026 PMC5456285 28933406

[6] Margine I Krammer F Hai R Heaton NS Tan GS Andrews SA. Hemagglutinin Stalk-Based Universal Vaccine Constructs Protect Against Group 2 Influenza A Viruses. J Virol (2013) 87:10435–46. 10.1128/JVI.01715-13 PMC3807421 23903831

[7] Coronaviridae Study Group of the International Committee on Taxonomy of V. The Species Severe Acute Respiratory Syndrome-Related Coronavirus: Classifying 2019-Ncov and Naming it SARS-CoV-2. Nat Microbiol (2020) 5:536–44. 10.1038/s41564-020-0695-z PMC7095448 32123347

[8] Lan J Ge J Yu J Shan S Zhou H Fan S. Structure of the SARS-CoV-2 Spike Receptor-Binding Domain Bound to the Ace2 Receptor. Nature (2020) 581:215–20. 10.1038/s41586-020-2180-5 32225176

[9] Zost SJ Gilchuk P Case JB Binshtein E Chen RE Nkolola JP. Potently Neutralizing and Protective Human Antibodies Against SARS-CoV-2. Nature (2020) 584:443–9. 10.1038/s41586-020-2548-6 PMC7584396 32668443

[10] Rajendran K Krishnasamy N Rangarajan J Rathinam J Natarajan M Ramachandran A. Convalescent Plasma Transfusion for the Treatment of Covid-19: Systematic Review. J Med Virol (2020) 92:1475–83. 10.1002/jmv.25961 PMC7267113 32356910

[11] Amanat F Stadlbauer D Strohmeier S Nguyen THO Chromikova V McMahon M. A Serological Assay to Detect SARS-CoV-2 Seroconversion in Humans. Nat Med (2020) 26(7):1033–6. 10.1101/2020.03.17.20037713 PMC8183627 32398876

[12] Millet JK Whittaker GR. Host Cell Proteases: Critical Determinants of Coronavirus Tropism and Pathogenesis. Virus Res (2015) 202:120–34. 10.1016/j.virusres.2014.11.021 PMC4465284 25445340

[13] Cameron A Porterfield CA Byron LD Wang J Pearson Z Bohrhunter JL. A Multiplex Microsphere IgG Assay for SARS-CoV-2 Using ACE2-Mediated Inhibition as a Surrogate for Neutralization. J Clin Microbiol (2021) 59(2):e02489–20. 10.1128/JCM.02489-20 33139422 PMC8111159

[14] Gillot C Douxfils J Cadrobbi J Laffineur K Dogne JM Elsen M. An Original ELISA-Based Multiplex Method for the Simultaneous Detection of 5 SARS-CoV-2 IgG Antibodies Directed Against Different Antigens. J Clin Med (2020) 9(11):2077–0383. 10.3390/jcm9113752 PMC7700260 33233405

[15] Weiss S Klingler J Hioe C Amanat F Baine I Arinsburg S. A High Through-Put Assay For Circulating Antibodies Directed Against The S Protein Of Severe Acute Respiratory Syndrome Coronavirus 2 (Sars-Cov-2). J Infect Dis (2020) 222(10):1629–34. 10.1093/infdis/jiaa531 PMC7499578 32860510

[16] Ayouba A Thaurignac G Morquin D Tuaillon E Raulino R Nkuba A. Multiplex Detection and Dynamics of IgG Antibodies to SARS-CoV2 and the Highly Pathogenic Human Coronaviruses SARS-CoV and MERS-CoV. J Clin Virol (2020) 129:104521. 10.1016/j.jcv.2020.104521 32623350 PMC7308014

[17] Wang J Hilchey SP DeDiego M Perry S Hyrien O Nogales A. Broad Cross-Reactive IgG Responses Elicited by Adjuvanted Vaccination With Recombinant Influenza Hemagglutinin (rHA) in Ferrets and Mice. PloS One (2018) 13:e0193680. 10.1371/journal.pone.0193680 29641537 PMC5894995

[18] Wang J Li D Wiltse A Emo J Hilchey SP Zand MS. Application of Volumetric Absorptive Microsampling (VAMS) to Measure Multidimensional Anti-Influenza IgG Antibodies by the Mplex-Flu Assay. J Clin Transl Sci (2019) 3:332–43. 10.1017/cts.2019.410 PMC6885997 31827907

[19] Wang J Li D Perry S Hilchey SP Wiltse A Treanor JJ. Cross-Reactive Anti-Hemagglutinin (HA) IgG Responses are Shaped by Previous Long-Interval Monovalent H5 Vaccination and Highly Correlated With HA Antigenic Distance. bioRxiv (2020). 10.1101/2020.10.14.340448

[20] Li D Wang J Garigen J Treanor JJ Zand MS. Broadly Reactive IgG Responses to Heterologous H5 Prime-Boost Influenza Vaccination Are Shaped by Antigenic Relatedness to Priming Strains. mBio (2021) e0044921. 10.1128/mBio.00449-21 34225490 PMC8406322

[21] Wang J Hilchey SP Hyrien O Huertas N Perry S Ramanunninair M. Multi-Dimensional Measurement of Antibody-Mediated Heterosubtypic Immunity to Influenza. PloS One (2015) 10:e0129858. 10.1371/journal.pone.0129858 26103163 PMC4478018

[22] Huang J Hilchey SP Wang J Gerigan J Zand MS. IL-15 Enhances Cross-Reactive Antibody Recall Responses to Seasonal H3 Influenza Viruses *In Vitro* . F1000Res (2017) 6:2015. 10.12688/f1000research.12999.1 29479423 PMC5801566

[23] Tesini BL Kanagaiah P Wang J Hahn M Halliley JL Chaves FA. Broad Hemagglutinin-Specific Memory B Cell Expansion by Seasonal Influenza Virus Infection Reflects Early-Life Imprinting and Adaptation to the Infecting Virus. J Virol (2019) 93(8):e00169–19. 10.1128/JVI.00169-19 PMC6450111 30728266

[24] Li D Wang J Treanor JJ Zand MS. Improved Specificity and False Discovery Rates for Multiplex Analysis of Changes in Strain-Specific Anti-Influenza Igg. Comput Math Methods Med (2019) 2019:3053869. 10.1155/2019/3053869 31178920 PMC6501432

[25] Sliepen K van Montfort T Melchers M Isik G Sanders RW. Immunosilencing a Highly Immunogenic Protein Trimerization Domain. J Biol Chem (2015) 290:7436–42. 10.1074/jbc.M114.620534 PMC4367253 25635058

[26] Walls AC Park YJ Tortorici MA Wall A McGuire AT Veesler D. Structure, Function, and Antigenicity of the SARS-CoV-2 Spike Glycoprotein. Cell (2020) 181:281–292 e6. 10.1016/j.cell.2020.02.058 32155444 PMC7102599

[27] Spooner N Denniff P Michielsen L De Vries R Ji QC Arnold ME. A Device for Dried Blood Microsampling in Quantitative Bioanalysis: Overcoming the Issues Associated Blood Hematocrit. Bioanalysis (2015) 7:653–9. 10.4155/bio.14.310 25514576

[28] Chan CM Woo PC Lau SK Tse H Chen HL Li F. Spike Protein, S, of Human Coronavirus HKU1: Role in Viral Life Cycle and Application in Antibody Detection. Exp Biol Med (Maywood) (2008) 233:1527–36. 10.3181/0806-RM-197 18849544

[29] Zand MS Wang J Hilchey S. Graphical Representation of Proximity Measures for Multidimensional Data: Classical and Metric Multidimensional Scaling. Math J (2015) 17:1047–5974. 10.3888/tmj.17-7 PMC4675631 26692757

[30] Ng KW Faulkner N Cornish GH Rosa A Harvey R Hussain S. Preexisting and De Novo Humoral Immunity to SARS-CoV-2 in Humans. Science (2020) 370(6522):1339–43. 10.1126/science.abe1107 PMC7857411 33159009

[31] Nguyen-Contant P Embong AK Kanagaiah P Chaves FA Yang H Branche AR. S Protein-Reactive IgG and Memory B Cell Production After Human SARS-CoV-2 Infection Includes Broad Reactivity to the S2 Subunit. mBio (2020) 11(5):2150–7511. 10.1128/mBio.01991-20 PMC7520599 32978311

[32] Wrobel AG Benton DJ Xu P Roustan C Martin SR Rosenthal PB. SARS-CoV-2 and Bat RaTG13 Spike Glycoprotein Structures Inform on Virus Evolution and Furin-Cleavage Effects. Nat Struct Mol Biol (2020) 27:763–7. 10.1038/s41594-020-0468-7 PMC7610980 32647346

[33] Grifoni A Sidney J Zhang Y Scheuermann RH Peters B Sette A. A Sequence Homology and Bioinformatic Approach Can Predict Candidate Targets for Immune Responses to SARS-CoV-2. Cell Host Microbe (2020) 27:671–680 e2. 10.1016/j.chom.2020.03.002 32183941 PMC7142693

[34] Shang B Wang XY Yuan JW Vabret A Wu XD Yang RF. Characterization and Application of Monoclonal Antibodies Against N Protein of SARS-Coronavirus. Biochem Biophys Res Commun (2005) 336:110 – 117. 10.1016/j.bbrc.2005.08.032 16112641 PMC7092910

[35] Dobano C Santano R Jimenez A Vidal M Chi J Rodrigo Melero N. Immunogenicity and Crossreactivity of Antibodies to the Nucleocapsid Protein of Sars-Cov-2: Utility and Limitations in Seroprevalence and Immunity Studies. Transl Res (2021) 232:60–74. 10.1016/j.trsl.2021.02.006 33582244 PMC7879156

[36] Dijkman R Jebbink MF Gaunt E Rossen JW Templeton KE Kuijpers TW. The Dominance of Human Coronavirus Oc43 and Nl63 Infections in Infants. J Clin Virol (2012) 53:135–9. 10.1016/j.jcv.2011.11.011 PMC7108278 22188723

[37] Zeng ZQ Chen DH Tan WP Qiu SY Xu D Liang HX. Epidemiology and Clinical Characteristics of Human Coronaviruses Oc43, 229e, Nl63, and Hku1: A Study of Hospitalized Children With Acute Respiratory Tract Infection in Guangzhou, China. Eur J Clin Microbiol Infect Dis (2018) 37:363–9. 10.1007/s10096-017-3144-z PMC5780525 29214503

[38] Mathew D Giles JR Baxter AE Oldridge DA Greenplate AR Wu JE. Deep Immune Profiling of Covid-19 Patients Reveals Distinct Immunotypes With Therapeutic Implications. Science (2020) 369(6508):1095–9203. 10.1126/science.abc8511 PMC7402624 32669297

