# Supplementary material for: Antibody Mediated Immunity to SARS-CoV-2 and Human Coronaviruses: Multiplex Beads Assay and Volumetric Absorptive Microsampling to Generate Immune Repertoire Cartography
Source: Front Immunol. 2021 Jul 27;12:696370. doi: 10.3389/fimmu.2021.696370 (PMC8353270; doi:10.3389/fimmu.2021.696370)
Supplement: Supplementary file 1 [file DataSheet_1.pdf]

frontiers

Supplementary Material

1 MATERIAL INFORMATION

1.1 Demographic information of subjects in this study

In this study, we enrolled three study cohorts: Pre-COVID-19, COVID-19 convalescents, and post-vaccination. The detailed information of subjects is listed in Supplementary Table S1.

Table S1. The demographic information of three cohorts in this study

		Pre-CoV	Post-COVID-19 *	COVID-Vac**
Number of Subjects (N)		21	18	15
Gender	F	14	12	9
	M	7	6	6
Age	Median	50	53	57
	Ave	46.5	51.4	50.1
Time after Infection or vaccination	Shortest	-	7	3
	Longest	-	1	179
	Ave	-	4.1	24.8
	Median	-	5	12.0

$*$ Months after COVID-19 diagnosis with RT-PCR.
$**$ Days after second injection of Pfizer COVID-19 vaccination.

1.2 Human coronavirus specific antibodies used in this study

We used commercial polyclonal and monoclonal antibodies to verify the bead coupling and mPlex-CoV assay, which are listed in Supplementary Table S2.

Table S2. The human coronavirus specific polyclonal or monoclonal antibodies in this study

Name	Type	Strain	Antigen Domain	Cat #	Dilution
mAb01	Chimeric MAb	SARS2	RBD	40150-D001	6400
mAb06	Chimeric MAb	SARS2	RBD	40150-D006	6400
mAb04	Chimeric Mab	SARS2	RBD	40150-D004	6400
SARS2 S1	Rabbit MAb	SARS2	S1	40150-R007	6400
SARS1 S1	Rabbit PAb	SARS1	S1	40150-T62-CoV2	1600
SARS1 S1	Rabbit PAb	SARS1	S1	40150-T62-50	6400
SARS2 RBD	Rabbit PAb	SARS2	RBD	40592-T62	6400
SARS2 S2	Rabbit PAb	SARS2	S2	40590-T62	6400
HKU1 S	Rabbit PAb	HKU1	S1+S2	40021-RP01	100
HKU1 S	Rabbit PAb	HKU1	S1+S2	40021-T60-50	1600
OC43 S1	Rabbit MAb	OC43	S1	ABIN2836774 *	80
MERS S1	Rabbit PAb	MERS	S1	100208-RP02	20
SARS2 N	Rabbit PAb	SARS2	N	40588-T62	6400
SARS1 N	Rabbit PAb	SARS1	N	40143-T62-50	6400
OC43 N	Rabbit MAb	OC43	N	MAB9013 **	6400

$*$ From antibodies-online
$**$ From emdmillipore.com.

2 SUPPLEMENTARY RESULTS

IgA and IgM antibody testing with mPlex-CoV assay. To estimate the concentration of antibodies against SARS-CoV-2 and other pathogenic human coronaviruses, we require a positive standard antibody control to create a standard curve for each virus strain, as previously described (Wang et al. (2019); Huang

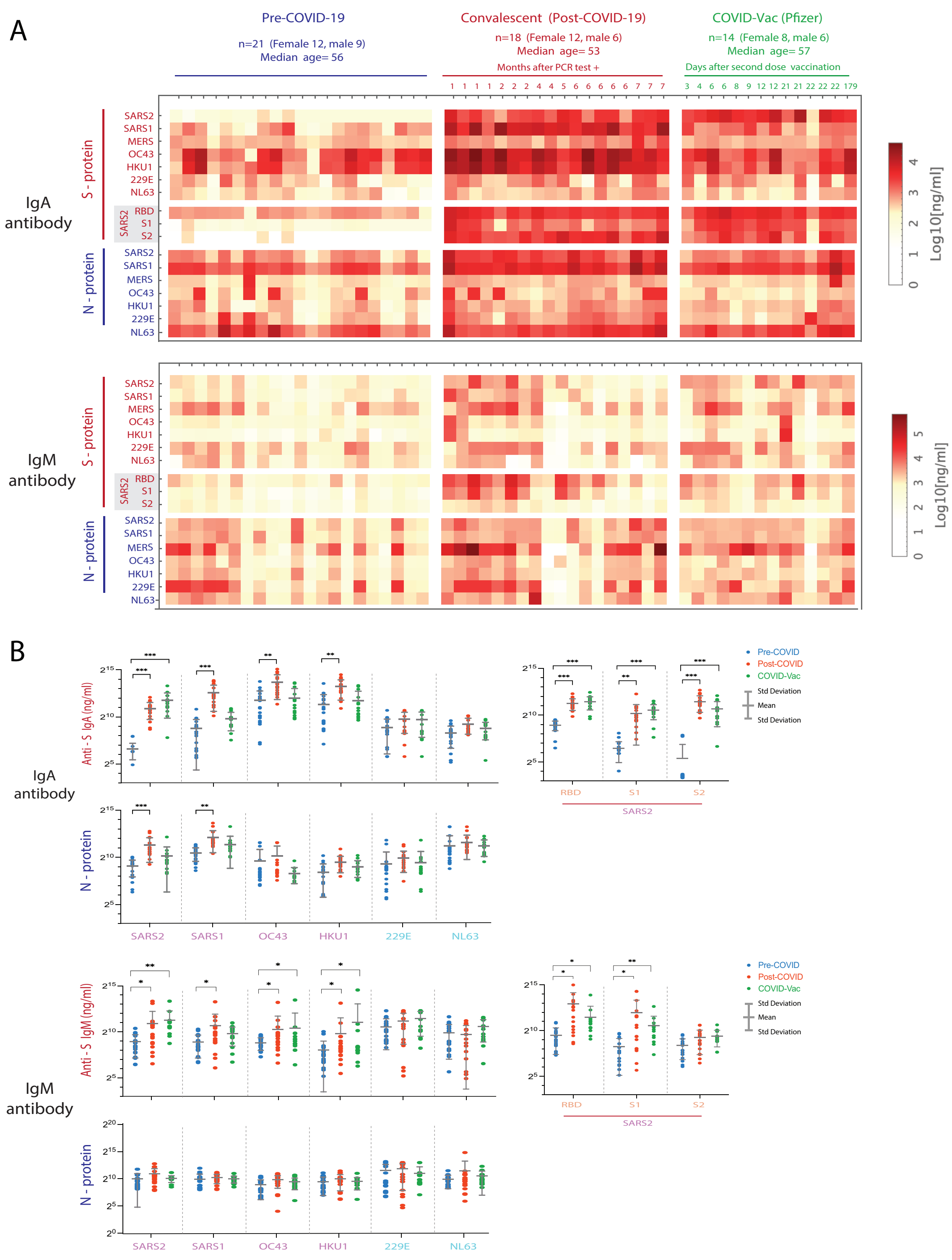

Figure S1. Spike (S)- and nucleocapsid (N)-reactive IgA and IgM antibody levels to human coronaviruses (HCoVs) in VAMS blood samples of pre-COVID, convalescents, and post-vaccination subjects. (A) The IgG concentrations (ng/mL) of each S and N-reactive IgA and IgM antibodies against HCoVs. The heatmap shows mean concentration (ng/ml) of duplicate samples. (B) Comparison of differences in range of anti-HCoV S- and N- protein IgA and IgM between pre-, post-COVID and post-vaccination cohorts. (*** p <0.001, ** p <0.01,,* p <0.05).

Table S3. The mPlex-CoV assay panel of human coronaviruses

Type	Coronavirus	Protein	Abbreviation region	Intra-assay CV(%)	Inter-assay CV(%)	ULOQ (ng/ml)	LLOQ (ng/ml)
β	SARS-Cov-2	S Protein	SARS2	3.51±1.12	9.6±5.92	146.32	0.16
	SARS-Cov-1		SARS1	3.77±2.24	9.95±5.33	16.37	0.49
	hCovOC43		OC43	3.16±1.88	6.13±3.95	78.53	0.99
	hCovHKU1		HKU1	3.16±1.13	6.77±5.58	57.92	0.817
α	hCov229E		229E	2.16±0.92	5.9±3.46	3.11	0.14
	hCovNL63		NL63	5.56±2.98	8.61±5.16	2.1	0.75
β	SARS-Cov-2	N Protein	SARS2	2.93±2.49	12.19±6.38	289.34	3.04
	SARS-Cov-1		SARS1	13.47±24.35	10.31±6.81	43.51	6.04
	hCovOC43		OC43	13.68±16.78	11.31±6.44	2.68	0.99
	hCovHKU1		HKU1	5.87±3.61	15.85±9.61	14.12	1.63
α	hCov229E		229E	5.99±4.54	8.82±5.6	10.98	1.07
	hCovNL63		NL63	13.13±19.67	6.22±3.31	31.42	10.54
β		SARS-CoV-2	RBD*	14±25.76	13.02±7.85	38.58	1.37
			S1*	2.56±2.23	13.69±5.52	116.5	1.23
			S2*	4.69±1.5	13.7±6.55	68.59	1.01

Table S4. The mPlex-CoV assay panel of human coronaviruses

Type	Coronavirus	Protein	Abbreviation region	Intra-assay CV(%)	Inter-assay CV(%)	ULOQ (ng/ml)	LLOQ (ng/ml)
β	SARS-Cov-2	S Protein	SARS2	2.52±2.46	7.5±4.66	196.56	0.26
	SARS-Cov-1		SARS1	3.97±3.76	6.34±5.3	15.83	0.72
	hCovOC43		OC43	3.44±3.01	5.38±4.41	12.12	1.42
	hCovHKU1		HKU1	11.07±4.21	6.88±5.97	13.91	1.26
α	hCov229E		229E	7.92±5.43	5.45±5.66	9.59	0.93
	hCovNL63		NL63	6.37±5.63	8.02±7.42	5.31	0.24
β	SARS-Cov-2	N Protein	SARS2	6.63±4.04	8.79±6.9	100.1	0.09
	SARS-Cov-1		SARS1	3.32±2.69	9.19±7.72	86.79	0.37
	hCovOC43		OC43	7.29±5.26	8.17±6.55	5.77	1.4
	hCovHKU1		HKU1	6.81±6.8	8.92±8.06	16.51	0.08
α	hCov229E		229E	1.88±1.41	7.68±5.14	5.97	0.07
	hCovNL63		NL63	7.46±3.62	7.63±6.6	8.94	0.13
β		SARS-CoV-2	RBD*	11.2±6.3	8.69±8.2	107.11	0.05
			S1*	8.02±8.33	7.17±5.36	44.47	0.05
			S2*	10.08±7.97	9.39±7.58	37.71	0.045

et al. (2017)). We generated a human coronavirus standard, STD-CoV, a mixture of four COVID-19 patient sera contain high levels of antibodies against most HCoVs. Furthermore, we used it to generate IgA and IgM standard curves for each HCoV virus strain and calculated the IgA and IgM antibody concentrations as it had been described in the method section.

REFERENCES

Wang J, Li D, Wiltse A, Emo J, Hilchey SP, Zand MS. Application of volumetric absorptive microsampling (VAMS) to measure multidimensional anti-influenza IgG antibodies by the mplex-flu assay. *J Clin Transl Sci* **3** (2019) 332–343. doi:10.1017/cts.2019.410.

Huang J, Hilchey SP, Wang J, Gerigan J, Zand MS. Il-15 enhances cross-reactive antibody recall responses to seasonal h3 influenza viruses in vitro. *F1000Res* **6** (2017) 2015. doi:10.12688/f1000research.12999.1.
